# An Update on Tamoxifen and the Chemo-Preventive Potential of Vitamin E in Breast Cancer Management

**DOI:** 10.3390/jpm13050754

**Published:** 2023-04-28

**Authors:** Farid Khallouki, Lhoussain Hajji, Somayya Saber, Toufik Bouddine, Mouad Edderkaoui, Mohammed Bourhia, Nora Mir, Adrian Lim, Adil El Midaoui, John P. Giesy, Mourad A. M. Aboul-Soud, Sandrine Silvente-Poirot, Marc Poirot

**Affiliations:** 1 Biology Department, FSTE, Moulay Ismail University of Meknes, BP 609, Errachidia 52000, Moroccosaber.somayya@gmail.com (S.S.);; 2 Biology Department, Faculty of Sciences, Moulay Ismail University of Meknes, BP. 11201 Zitoune, Meknes 50050, Morocco; 3 Departments of Medicine and Biomedical Sciences, Cedars-Sinai Medical Center & University of California, Los Angeles, CA 90048, USA; 4 Higher Institute of Nursing Professions and Technical Health, Laayoune 70000, Morocco; 5 Toxicology Centre, University of Saskatchewan, Saskatoon, SK S7N 5B3, Canada; 6 Department of Veterinary Biomedical Sciences, University of Saskatchewan, Saskatoon, SK S7N 5B4, Canada; 7 Department of Integrative Biology, Michigan State University, East Lansing, MI 48824, USA; 8 Department of Environmental Sciences, Baylor University, Waco, TX 76706, USA; 9 Medical and Molecular Genetics Research, Department of Clinical Laboratory Sciences, College of Applied Medical Sciences, King Saud University, P.O. Box 10219, Riyadh 11433, Saudi Arabia; 10 Cancer Research Center of Toulouse, UMR 1037 INSERM, UMR 5071 CNRS, University of Toulouse III, Equipe labellisée par la Ligue Nationale Contre le Cancer, 31037 Toulouse, France; 11 French Network for Nutrition And Cancer Research (NACRe Network), 78350 Jouy-en-Josas, France

**Keywords:** tamoxifen, estrogen receptor, AEBS, ChEH, cholesterol metabolism, cholesterol biosynthesis, oxytserols, breast cancer, cytotoxicity, autophagy, resistance

## Abstract

Breast cancer (BC) is the most common female cancer in terms of incidence and mortality worldwide. Tamoxifen (Nolvadex) is a widely prescribed, oral anti-estrogen drug for the hormonal treatment of estrogen-receptor-positive BC, which represents 70% of all BC subtypes. This review assesses the current knowledge on the molecular pharmacology of tamoxifen in terms of its anticancer and chemo-preventive actions. Due to the importance of vitamin E compounds, which are widely taken as a supplementary dietary component, the review focuses only on the potential importance of vitamin E in BC chemo-prevention. The chemo-preventive and onco-protective effects of tamoxifen combined with the potential effects of vitamin E can alter the anticancer actions of tamoxifen. Therefore, methods involving an individually designed, nutritional intervention for patients with BC warrant further consideration. These data are of great importance for tamoxifen chemo-prevention strategies in future epidemiological studies.

## 1. Introduction

Worldwide, breast cancer (BC) is one of the most common malignant neoplastic diseases and the second leading cause of cancer death in women. In 2018, more than two million individuals were diagnosed with BC and, unfortunately, 15% of them have since died [1]. In 2019, in the US, BC alone accounted for 30% of all new cancer diagnoses in women [2]. In 2020, female BC also surpassed lung cancer to become the most diagnosed cancer worldwide [3].

BC heterogeneity consists of the determination of histopathological biomarkers and gene expression profiling. Luminal A, Luminal B, HER2, and triple-negative/basal-like are the four main subtypes of breast cancer. Luminal A, HER2-positive BC, and basal-like BC are also associated with worse prognoses [4,5,6,7].

ER+ BC represents the major subclass of BC, which can either be complementary, palliative, or inductive, and accounts for 70–75% of all BC [6,8]. The hormone therapy of BC was the first targeted therapy of tamoxifen (TAM), with the chemical name (*Z*)-2-[4-(1,2-diphenylbut-1-enyl) phenoxy]-*N*,*N*-dimethylethanamine. TAM, which belongs to a group of selective estrogen receptor modulators (SERMs), is an old and widely prescribed prodrug that is utilized worldwide for the treatment of BC. To this day, TAM remains the standard and complementary endocrine therapy to surgery. It is used in chemo-prevention for patients with estrogen receptor α (ERα)-positive and ER-negative BC cells.

In addition, vitamins such as vitamin E are among the most frequently used supplements [9]. Furthermore, they are highly present in the “Mediterranean” diet of Western hominins. Vitamin E supplements have been an effective approach to improving quality of life and overcoming the side effects of TAM. In addition, due to its antioxidant nature, vitamin E might play an essential role in protecting the body against oxidative stress at the cellular level. However, several studies have shown evidence that vitamin antioxidant supplements might actually compromise treatments by protecting tumor cells during hormonal therapy, radiotherapy, and chemotherapy [10,11].

The biological activity of vitamin E and other antioxidants depends on several factors, including genetic predisposition, oxidative stress levels, antioxidant interactions, nutritional status, socio-demographic factors, lifestyle, dose, and bioavailability [12,13,14]. Moreover, cancer might cause profound metabolic disorders that could affect the metabolism of vitamin E and other nutrients. Therefore, the modern treatment of hormonal BC has become much more individualized, requiring interdisciplinary treatment. The rehabilitation of oncological patients focuses on a reduction in long-term adverse effects, pain management, nutritional treatment, physical activity, and the constant control of relapses.

This review will examine the comprehensive role of TAM in BC and its mechanisms of resistance. We will also provide an update on what has been learned regarding the potential role of vitamin E in BC patients, as well as discussing the possibilities for negating the effects on TAM interaction during hormonal BC management.

## 2. The Literature Search

To compile research about breast cancer, instances of tamoxifen (TAM) and vitamin E that were taken simultaneously or separately, and the use of electronic databases—including PubMed and Google Scholar—were searched for relevant papers. The search terms were assessed solely or in the combinations of “tamoxifen and breast cancer chemo-prevention”, “tamoxifen and antiestrogen binding site”, “acquired resistance and tamoxifen”, and “tamoxifen adverse effects”, as well as “Vitamin E esters, tocotrienols and cancer chemo-prevention”. For the search results of the concomitant treatment of vitamin E and tamoxifen, the operator “AND” was added to help to downsize the search results, as well as for the purposes of including publications from their inceptions to date: “tamoxifen and vitamin E and oxidative stress” and “tamoxifen and vitamin E and breast cancer chemo-prevention”. The synonyms of vitamin E (tocopherols or tocotrienols) were also used as related terms to locate further potential evidence to be included in this review. Observational, randomized controlled trials, in vivo and in vitro studies, and epidemiological studies published in English were all examined.

## 3. A Brief Description of Tamoxifen and Its Interaction with the ER

Estrogens, primarily produced in the ovarian follicles and adrenal glands, are the most important female sex hormones [15]. The estrogen receptor (ER) is a member of the nuclear receptor superfamily; its function, which is mediated by 17β-estradiol (E2), is to activate the transcription of genes involved in the growth and differentiation of cells, as well as reproduction.

The two distinct subtypes of ER, ERα and ERβ, each have different effects in gene regulation and cell proliferation. ERα is mainly present in tissues with reproductive functions, such as the ovaries, uterus, and breast, along with the brain, heart, liver, and bone. ERβ is detected in the ovarian, uterine, breast, hypothalamic, and cardiovascular tissues [16].

**Figure 1:**
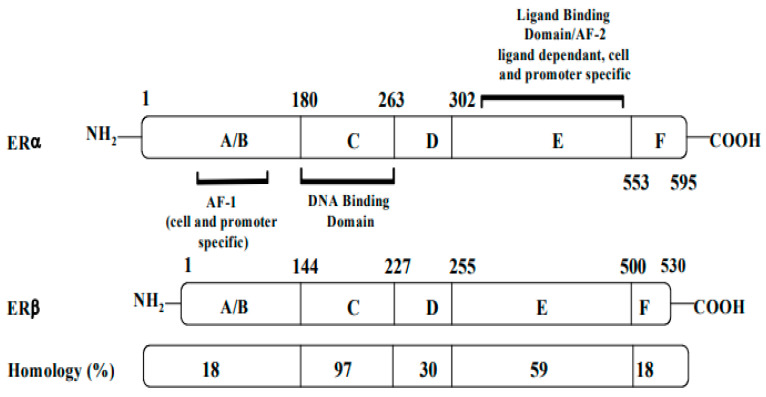
Structures, functional regions, and percentage of sequence homology of two human estrogen receptors: ERα and ERβ.

Structurally, ERs consist of six functional domains: A, B, C, D, E, and F, with three major domains including an *N*-terminal (A/B domain), a DNA-binding domain (C domain), and a ligand-binding domain (LBD, E domain) (Figure 1). The *N*-terminal (A/B domain) activation function (AF-1) region is involved in protein–protein interactions, is hormone-independent, and is mostly regulated by phosphorylation. The C-terminal (E domain) activation function (AF-2) contains the binding region for the ligand, along with co-activators or co-repressors, and is responsible for ligand-gated transcription. The highly conserved DNA-binding C domain consists of two zinc finger modules, which establishes contacts with specific response elements and is involved in the spatial recognition of the receptor. The flexible hinge or D domain contains a nuclear localization signal, acting as a dimerization region. Lastly, the F domain is variable, and its function is still poorly understood [15].

Both receptors are organized in six functional domains: the A/B domain at the *N*-terminal, the C domain of the DNA-binding domain (DBD), the D domain of the nuclear translocation signal, and the E/F domain at the C-terminus including the ligand-binding domain (LBD) and the ligand-dependent activation function AF-2.

AF: activation function (taken and modified from reference [16]).

Although the two ERs possess different functions, they share a homologous DNA-binding domain and a 55–60% homology in their ligand-binding domains (LBDs). Both coexist in breast tissues, with more ERα than ERβ in breast tumors [17]. While AF-1 has only 20% homology in ERα and ERβ, AF-2 is especially similar in both ERs [18].

In the presence of natural or synthetic agonists of the ER, AF-1 and AF-2 domains will interact with steroid receptor coactivator proteins. These include the p160/steroid receptor coactivator (SRC) family, which comprises three pleiotropic coregulators (SRC-1, SRC-2, and SRC-3) that serve as the transcription factors that regulate the expression of genes [19,20]. Hormone-activated receptors form multiple dimers of αα monomers, ββ monomers, and a combination of αβ monomers [21]. Depending on the composition of the dimers, cell homeostasis regulation and the affinity for different estrogen response elements (EREs) might vary.

**Figure 2:**
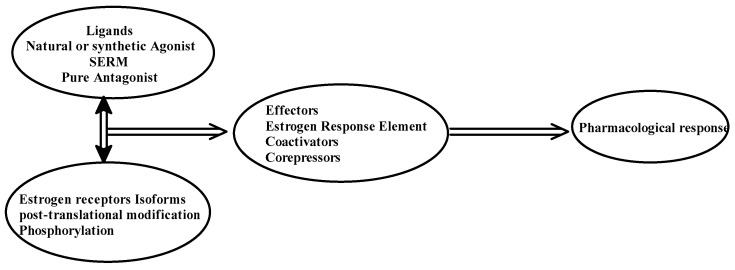
Pharmacological action of estrogen receptors.

The selective action of the ER might depend on the structure of the ligand, the type or isotype of ER, the type of co-regulator, and the interaction of the complex with EREs. Figure 2 depicts the action of ER in terms of explaining the tissue-specific binding of E2 to its receptors.

**Figure 3:**
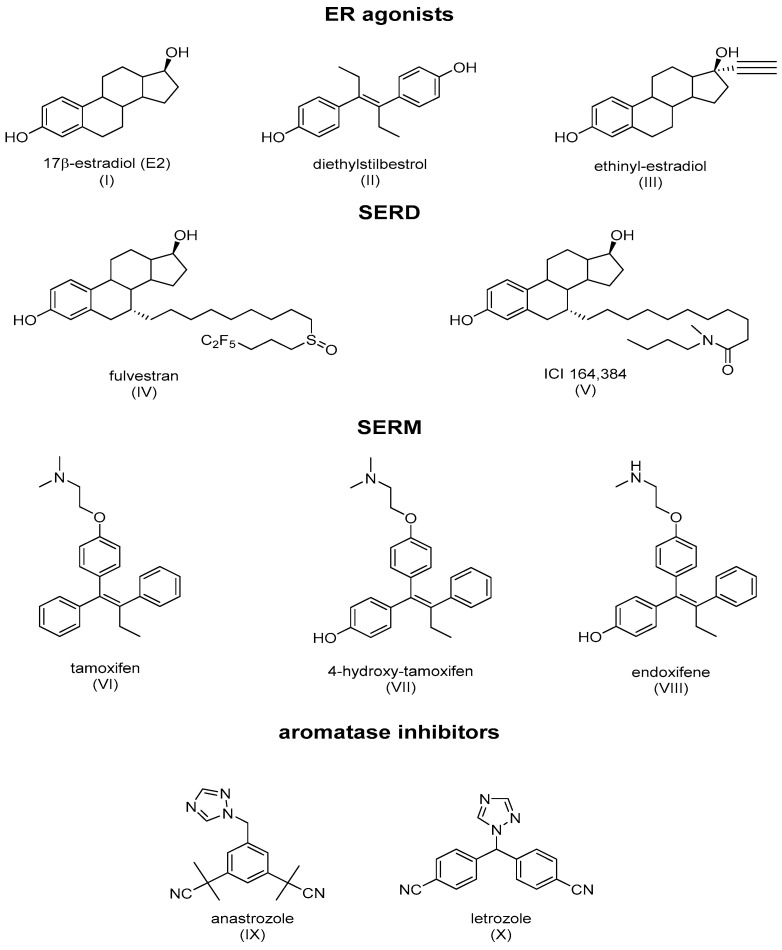
Main classes of agents used in hormone therapy that target estrogen signaling: ER agonists, SERDs (Fulvestran and ICI 164,384), SERMs (tamoxifen), and aromatase inhibitors.

ER ligands are classified into four families: ER agonists, SERDs, SERMs, and aromatase inhibitors (Figure 3). ER agonists include endogenous natural 17β-estradiol (Figure 3I), diethylstilbestrol (Figure 3II), and ethinylestradiol (Figure 3III). In addition, 17β-estradiol (E2) agonizes ERs in all tissue types where they are expressed and recruit coactivators to activate gene expression under the control of EREs, thereby promoting DNA synthesis and the proliferation of ER-responsive cells. Selective degraders of estrogen receptors (SERDs) completely inhibit and degrade ERα. Examples include the synthetic drugs ICI 182,780 (Fulvestrant) (Figure 3IV) and ICI 164,384 (Figure 3V), with the systematic name of *N*-n-butyl-*N*-methyl-11-[3,17β-dihydroxy-estra-1,3,5(10)-trien,7α-yl]-undecanamide. Selective modulators of estrogen receptors (SERMs) are unique in their anti-estrogen activity, depending on the type of tissue. Mounting evidence supports the unique characteristics of SERM activity, which is primarily determined by the selective recruitment of both ERα repressors and activators into specific tissues [18,22]. The main example is tamoxifen (TAM), which is a partial agonist of the first generation of SERMs (Figure 3VI). The final class of drugs, including anastrozole (Figure 3IX) and letrozole (Figure 3X), are aromatase inhibitors (AIs). AIs inhibit the aromatization of androgens at the CYP9A1 level, and block the endogenous formation of estrogens.

Dr. Craig V. Jordan gave experimental proof that TAM blocks the mitogenic action of 17β-estradiol and displays chemo-preventive properties in a chemically induced mammary carcinoma rat model [23]. These data contributed to approval by the Food and Drug Administration (FDA) of TAM for its clinical use in 1977. The recommended daily dose of TAM in the treatment of BC is currently 20 mg. TAM was found to be metabolized in the liver by flavin-containing monooxygenase and cytochrome P450 enzymes, mostly CYP2D6 and CYP3A4/3A5, giving rise to a 30–100-fold increase in affinity to ERα for endoxifen (Figure 3VIII) and 4-hydroxytamoxifen (Figure 3VII) [24,25].

TAM acts by blocking the mitogenic action of E2, via competition with E2 on ERα [26,27]. The structural analyses of ERα-4OHTam were conducted and showed that the drug effectively accommodates the ER ligand-binding pocket [28,29,30]. OH-TAM induced conformational changes that were different from E2 on ER-LBD, which affected the recruitment of the co-regulators and the regulation of gene expression [31]. The LBD, which consists of amino acids 304 to 553 of the ER, is composed of a cluster of twelve α-helices (H1–H12), which harbor a highly structured ligand-dependent domain containing dimerization interfaces, co-activation and co-repression functions, and a hydrophobic pocket that accommodates hormones.

ERα, in the absence of hormones, is inactivated as a complex by several chaperone proteins, such as HSP90, HSP70, and HSP40, with the aid of several other co-chaperones including cyclophilin-40 and p23 proteins. These heat shock proteins help to maintain the receptor in an adequate conformation, allowing for it to respond quickly to a hormonal signal. The binding of the hormone to the receptor results in the release of chaperonins and induces the nuclearization of the ER-E2 complex, which is followed by dimerization and stabilization in a conformation where the last helix (H12) folds over the ligand-binding pocket (LBP) and forms a hydrophobic groove, sealing the LBP [32].

**Figure 4:**
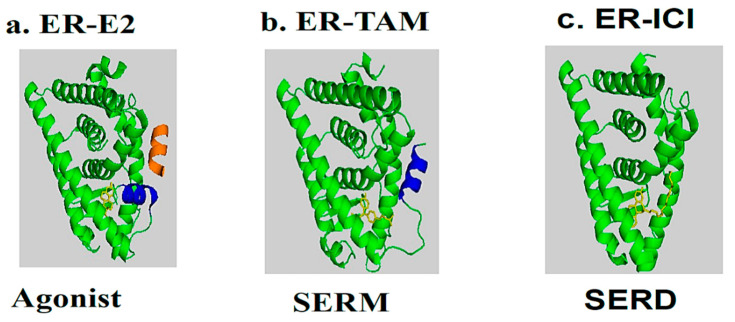
Molecular model and ribbon representation highlighting the action mechanism of estrogen receptors and the positioning of helix H12. (**a**) ER-E2 represents estradiol (yellow) in the presence of a co-activator (orange). (**b**) ER-TAM shows ER-LBD with tamoxifen (yellow), which is conducted to induce co-repression (SERM). (**c**) The ER-ICI interaction shows the full antagonist activity (SERD) in the absence of helix H12. Helix H12 in (**a**,**b**) is drawn as a cylinder in blue (Poirot et al., unpublished data).

In the presence of agonists, helix 12 traps the ligand inside the hydrophobic pocket of the ligand-binding domain. Helix 12’s hydrophobic grooves become exposed to the nuclear box recognition sequence containing the key LXXLL motif, which is rich in leucine. Alternatively, SERM drives the hydrophobic pocket into an open state, which dislocates helix 12 from forming an active AF-2 conformation, and specifically occupies the space for a coregulatory protein recognition sequence of LXXLL binding, thereby blocking the coactivators from binding and preventing gene transcription. The crystal structures of LBD complexed with 4-hydroxy-tamoxifen and ICI-164, 384 (Figure 3V) (pure antiestrogen) reveal evidence of certain critical parameters with regard to these interactions. Figure 4 depicts the mechanism of action of the ER ligands at the ERα level. E2, TAM, and ICI 164,384 (ICI) bind to ER LBD, which explains why they compete with E2 at the ERα level and can inhibit E2-mediated BC cell proliferation. TAM might exert its antiproliferative potency through other ER-dependent mitogenic pathways [33,34,35,36,37]. TAM also possesses ERα-independent antiproliferative properties and displays a certain degree of efficacy in the context of ER-negative cancer cells (see vide infra).

## 4. Insight to the Chemo-Preventive Action of Tamoxifen in Breast Cancer

Chemo-prevention is an oncological prophylaxis that modulates the process of carcinogenesis in the early stages of cancer development with the use of pharmacological agents and non-nutritional food components [38]. Since the 1970s, TAM has been a landmark form of BC treatment. It was initially recommended as an antiestrogen to help treat hormone-responsive BC, but its curative potential was later expanded to adjuvant therapy in order to help reduce the incidence of BC. TAM inhibits cell proliferation through its actions on the ERα, which regulates the processing of growth factors. This can lead to the arrest of cell growth, or cell death and tumor regression. TAM was, therefore, shown to be an efficient chemo-preventive agent in both postmenopausal and premenopausal women with a moderate risk of BC [39]. The results of meta-analyses of randomized clinical trials and case–control studies reported a decrease in BC incidence in women receiving TAM as a postoperative adjuvant compared to untreated women [6,40,41,42,43,44,45,46,47].

Adjuvant TAM is commonly administered for 5 years; however, 10 years of TAM therapy has been associated with a greater efficacy in patients with ER-positive BC. In addition, this longer duration of therapy might reduce mortality caused by BC during the second decade after diagnosis by half [46,48]. Moreover, TAM reduces the risk of developing ER-positive BC by 48% in women over 35 years old [49]. Although the main rationale in the clinical use of TAM is the blockage of mitogenic actions of E2, several other mechanisms of TAM have been observed that could account, in part, to its anticancer action. Furthermore, TAM has been shown to induce cell death, which has been earlier summarized in certain other excellent reviews [50,51,52].

**Figure 5:**
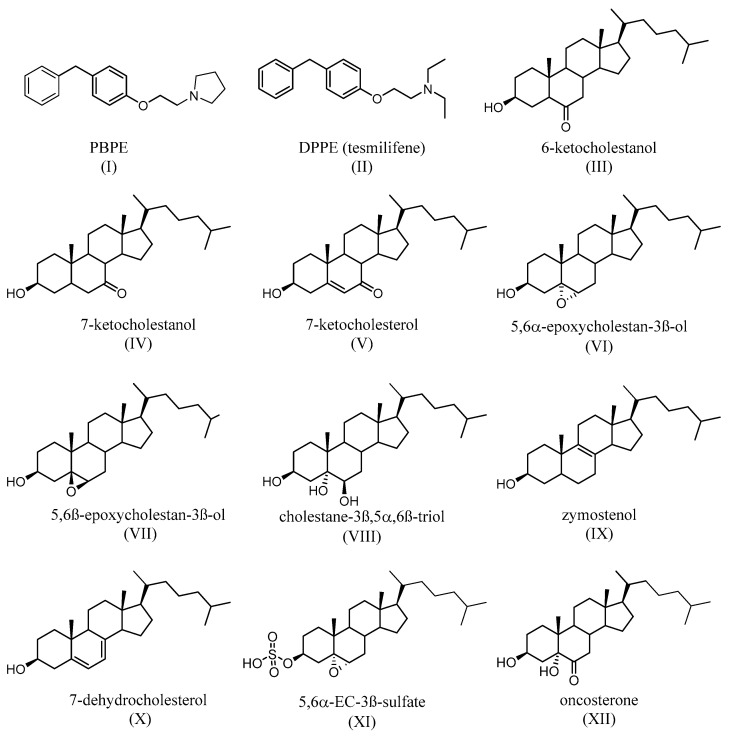
Synthetic (**I**,**II**) and endogenous (**III**–**V**) selective AEBS ligands with no affinity for ER. Oxysterols that accumulate after the inhibition of ChEH by AEBS ligands (**VI**–**VII**). Product of the ChEH enzymatic activity (**VIII**). Cholesterol precursors that can accumulate after the binding of ligands (including SERMs) on the AEBS (**IX**,**X**). The signaling sterol that accumulates in cells expresses SULT2B1b, after ChEH inhibition by AEBS ligands (**XI**). The tumor promoter whose biosynthesis is blocked by AEBS ligands (including SERMs) (**XII**).

TAM binds with a nanomolar affinity to a microsomal binding site, called the anti-estrogen binding site (AEBS) [53]. Selective AEBS ligands include the diphenyl methane compounds PBPE (Figure 5I) and DPPE (tesmilifene, Figure 5II). AEBS ligands include SERMs, bearing a cationic amino alkyl side chain; estrogens and pure antiestrogens, on the other hand, have no affinity for the AEBS. Oxysterols (e.g., 6-ketocholestanol (Figure 5III), 7-ketocholestanol (Figure 5VI), and 7-ketocholesterol (Figure 5V), as well as unsaturated fatty acids and histamine), have also been characterized as AEBS ligands [51]. AEBS is found in various tissues, including BC cells, and is independent of ER status. Kedjouar et al. reported that AEBS is a multiproteic complex composed of two cholesterogenic enzymes: 3β-hydroxysterol-Δ8-Δ7-isomerase (EBP/D8D7I) and 3β-hydroxysterol-Δ7-reductase (DHCR7) [54]. It was later shown that the AEBS carries out cholesterol-5,6-epoxide hydrolase (ChEH) enzymatic activity that catalyzes the hydrolysis of 5,6-epoxycholestan-3β-ol diastereomers (5,6α-EC (Figure 5VI) and 5,6β-EC (Figure 5VII) into cholestane-3β,5α,6β-triol (CT) (Figure 5VIII) [55,56].

TAM, SERMs, and the selective AEBS ligand PBPE (Figure 5I) induce the intracellular accumulation of cholesterol precursors, including zymostenol (Figure 5IX), the substrate of D8D7I, and 7-dehydrocholesterol (Figure 5X), which is the substrate of DHCR7. AEBS ligands also increase the total sterol levels in the cell [57]. Interestingly, accumulated cholesterol precursors undergo oxidation toward unidentified oxysterols. Since AEBS also carries out ChEH activity, the intracellular accumulation of 5,6-EC and the diminution of CT (Figure 5VIII) have thus been described, on BC cells, as being exposed to AEBS ligands. In addition, CT is metabolized by 11β-hydroxysteroid-dehydrogenase type 2 into oncosterone (Figure 5XII), an oncometabolite that promotes BC development [58]. Consequently, targeting ChEH with AEBS ligands inhibits both the production of CT and oncosterone in BC cells. It was shown that the inhibition of oncosterone production represents a promising approach for the development of new anticancer agents [58,59,60].

5,6α-EC that is produced and accumulated in an ER-positive BC cell line (MCF-7) treated with TAM was shown to be transformed into 5,6α-EC-3β-sulfate (CES, Figure 5XI) via steroid sulfotransferase SULT2B1b. Importantly, it was shown that CES was the signaling molecule induced by the AEBS ligands in MCF-7. This is responsible for the cell differentiation and death that is induced by AEBS ligands, including SERMs, in an LXRβ-dependent manner. This was not observed in the ER-negative BC cell line MDA-MB-231, which does not express SULT2B1b [61] and was previously described to be intrinsically resistant to TAM [62]. Interestingly, the ectopic expression of SULT2B1b sensitized these cells to AEBS ligands to the same level that is seen in MCF-7 cells [61].

In summary, AEBS ligands profoundly affect cholesterol metabolism through two mechanisms: (1) they induce the intracellular accumulation of cholesterol precursors and their auto-oxidation products, and (2) they stimulate the epoxidation of cholesterol and the accumulation of 5,6-EC due to the inhibition of ChEH. Furthermore, 5,6-EC and the sulfated CES are responsible for the cell death and differentiation induced by AEBS ligands. In addition, AEBS ligands drastically decrease the level of the oncometabolite oncosterone in BC cells [58].

## 5. Acquired Resistance to Tamoxifen

Unfortunately, within a few years, almost all responsive patients eventually develop acquired resistance [63]. The development of resistance to the effects of drugs remains a major obstacle in hormonal therapy with TAM. Resistance to the effects of TAM might be the result of various mechanisms that might be linked to high levels, losses, or alterations in the ER. A total of 30% of ER-positive tumors acquire resistance to TAM [64]. Compared to their more treatment-resistant HER2-positive counterparts, ER-negative and -positive patients responded well to treatment with TAM, regardless of their progesterone status [65]. Additionally, the ER can be active in cell membranes. When bound to estrogen or SERMs, it could trigger cellular proliferation, where TAM acts as an agonist [66,67]. An example of a mechanism underlying resistance is the intricate interaction of surface receptors, especially G-protein-coupled receptors (GPCRs) [68]. It has been demonstrated that G-protein-coupled estrogen receptors (GPERs) mediate multiple estrogenic signals in various types of malignant cells [69] and respond to estrogen via the up-regulation of aromatase (also called estrogen synthetase or estrogen synthase, CYP19A1), the adrenal enzyme that converts androstenedione and estrone to estrogen (T) [70]. More particularly, GPR30 was detected in nearly 62% of invasive tumors and was co-expressed with the ER in about 43% of BC cases. Furthermore, the expression of GPR30 is inversely correlated with the expression of ER [71] and also attenuates the inhibition of mitogen-activated protein kinases (MAP kinases), thereby contributing to resistance to TAM in BC [72,73]. Resistance to TAM might also take place through growth factors, survival factors, and chemokines, which support tumorigenesis. Such signaling, which includes HER-2/neu, TGFβ, Notch, and the insulin-like growth factor receptor, demonstrates multiple pathophysiological functions associated with oncogenic kinase signal transduction pathways, such as the phosphatidylinositol3-kinase (PI3K)/Akt/mammalian target of the rapamycin (mTOR) signaling pathway (PI3K/Akt/mTOR) and the Ras/Raf/MEK/ERK axis [66,74,75,76,77,78,79,80,81,82].

In breast cancer cells, TAM up-regulates mRNA for transforming growth factor-beta (TGF-β2) with no correlation with the status of αER and PR [75]. TGF-β2 is a secreted protein known as a cytokine that can be involved in various cellular functions. Specifically, in BC, TAM inhibits the TGF-β-mediated activation of breast fibroblasts. TAM blocks non-SMAD signaling through ERK1/2 MAP-kinase and the transcription factor FRA2 [82]. Therefore, TAM might provide therapeutic benefits by inhibiting the differentiation of myofibroblasts. Myofibroblasts are involved in promoting the growth and invasiveness of tumors [83]. However, the differentiation and activation of myofibroblasts have been observed in other cancers, such as pancreatic cancer, where they contribute to the prevention of the development of tumors; thus, the antimyofibroblast effect of TAM might cause chemoresistance in BC [84]. Moreover, TGFβ might also promote epithelial-mesenchymal transition (EMT) and cell invasiveness, depending on the cell context, growth factor environment, and stage of BC [85]. The delineation of the other molecular aspects associated with resistance to the therapeutic effects of TAM includes impaired metabolism, especially by CYP2D6 [86]. Certain CYP2D6 variants were reported to diminish clinical outcomes in patients treated with TAM [87,88].

The effect of TAM on metabolism involving glucose is another important mediator in the resistance of cancer cells to TAM. Glucose transporter 1 (GLUT1) plays a role in increasing autophagy and in acquiring resistance to TAM in BC cells [89]. In fact, the overexpression of GLUT1 has been observed in aggressive breast cancer and is correlated with a poor prognosis for BC patients [90]. During the development of resistance of BC cells to TAM, the activation of protein kinase B, also known as AKT kinase, and the decreased levels of AMPK lead to the activation of hypoxia-inducible factor 1α (HIF-1α). AKT is the collective name for a set of three serine/threonine-specific protein kinases that are involved in various processes in cells, including glucose metabolism, apoptosis, proliferation, transcription, and the migration of cells. The development of resistance to TAM was found to be driven by hypoxia-inducible factor-1 alpha HIF-1α, making the AKT and AMPK pathways good targets for overcoming resistance to TAM [91]. (HIF-1α) regulates the responses of cells to oxygen concentration, supporting the adaptation of tumor cells to hypoxia in the oxygen-deficient microenvironment of rapidly growing tumors. Thus, HIF-1α is important in carcinogenesis and progression tumors. HIF-1α is associated with a poor prognosis in BC patients [92].

A recent study reported that lactate dehydrogenase A (LDHA) induces pro-survival autophagy, thereby leading to the resistance of BC cells to TAM. LDHA, in association with Beclin 1, induces autophagy, thereby causing the inhibition of TAM-induced apoptosis. Thus, LDHA is another potentially good target for preventing resistance to TAM in BC [93].

TAM accumulates in the mitochondria and can affect multiple mitochondrial functions. TAM inhibits oxidative phosphorylation and fatty acid oxidation by binding to the Flavin mononucleotide molecule of complex-I, thus resulting in mitochondrial electron transport chain dysfunction [94]. Cells resistant to TAM are characterized by greater glycolysis and exhibit increased AMPK phosphorylation and the activation of mitochondrial protein deacetylase, which is known as Sirtuin 3 (SIRT3) [95,96].

TAM might behave as an estrogen agonist in BC cells that express greater amounts of human epidermal growth factor receptor 2 (HER2), a protein that promotes the growth of cancer cells, resulting in de novo resistance [97]. TAM might bind and activate ERα36—a variant of ERα in BC stem cells that is associated with poor prognoses—in order to enhance the stemness and metastasis of BC cells via the transcriptional stimulation of the protein-coding gene Aldehyde Dehydrogenase 1 Family Member A1 (ALDH1A1) [98]. In a more recent report, it was reported that oncogenic p21-activated kinase-1 (PAK1) might also reload resistance to TAM by phosphorylating ERα and other substrates in ER-positive BC patients [99]. Alternative mechanisms of resistance to TAM might also be due to the activation of inflammatory cytokines, such as interleukin-1 beta (IL-1β). IL1β is also known as leukocytic pyrogen, leukocytic endogenous mediator, mononuclear cell factor, and lymphocyte activating factor. Resistance to TAM might also be caused by mechanisms involving tumor necrosis factor and alpha (TNFα), which are associated with Nuclear Factor-Kappa B (NF-κB) [100].

BC inhibition might be mediated by nuclear factor erythroid-2 related factor-2 (Nrf2), which is a member of the cap ‘n’ collar (CNC) subfamily of the basic region leucine zipper (bZip) transcription factor. This acts as the master regulator controlling the expression of antioxidant genes that regulate physiological and pathophysiological events following exposure to oxidants. Under oxidative stress, the complex Kelch-like ECH-associated protein 1 (KEAP1), which is a subunit of CULLIN 3 (CUL3)-based E3 ubiquitin ligase, and Nrf2 dissociates, and Nrf2 is then translocated into the nucleus where it heterodimerizes with the small musculoaponeurotic fibrosarcoma proteins (MAF), which are basic region leucine zipper-type transcription factors that can bind to DNA and regulate gene regulation by binding to the antioxidant-responsive element (ARE) and stimulating the gene expression of antioxidants and detoxification enzymes. Consequently, the knockdown of Nrf2 increases TAM-induced cell death in TAM-resistant cells [101,102,103,104,105]. MicroRNAs (miRNAs), a class of short non-coding RNAs commonly involved in treatment regimens, were shown to play a pivotal role as regulators of various biological processes, such as the therapeutic targets involved in ERα regulation [104]. The mechanisms of resistance have been correlated with a change in the expression of miRNA, as well as the remodeling of the epithelial to mesenchymal transition (EMT) which is involved in the formation of tumors [105]. The expression of miR-221 and miR-222, encoded in tandem on the X chromosome, has been found to be elevated two-fold in endocrine-therapy-resistant HER2/neu-positive primary human BC tissues [106,107]. Although TAM is involved in the regulation of microRNAs, miR-221/222 has been found to confer resistance to TAM in BC by reducing the expression of the cell cycle regulator p27/kip1 [108].

Another possible contributor to the resistance is the microsomal antiestrogen binding site (AEBS). The ligands of the AEBS do not bind to ERs, but bind selective ER modulators (SERMs). AEBS ligands contain a hydrophobic core that mimics the steroid backbone of estrogens. The antitumor properties of AEBS ligands and their relationship with cholesterol metabolism perturbations have been discussed previously. AEBS ligands, including tesmilifene (Figure 5II) and PBPE (Figure 5I), display antitumor properties through their impact on cholesterol metabolism. Indeed, AEBS ligands, including SERMs, induce the redifferentiation of BC cells into lactating epithelial cells, which is characterized by a cell cycle arrest in the G0-G1 phase, morphological modifications of triglycerides, and the secretion of milk proteins [58,109,110]. BC cell redifferentiation results from the accumulation of CES and is mediated by liver-X-receptor β, an oxysterol-activated nuclear receptor [61]. Alternatively, the ligands of AEBS trigger a pro-survival autophagy that limits their antitumor properties via the accumulation of cholesterol precursors, such as zymostenol [109,110]. Additionally, antioxidant defense has been reported as a mechanism of resistance against TAM’s antitumor property for BC patients. Thus, these data afford a new rationale that involves a relationship between AEBS, cholesterol metabolism, oxidative stress/antioxidant defense, and responses to TAM.

TAM and *N*-pyrrolidino- (phenylmethyphenoxy)-ethanamine,HCl) (PBPE) trigger intracellular formation and the accumulation of 5,6α-EC and 5,6β-EC in BC cells through both the induction of oxidative stress and the inhibition of ChEH. In addition, 5,6α-EC is transformed into CES (Figure 5XI) in MCF-7 cells that express SULT2B1b but not in MDA-MB-231 cells that do not express SULT2B1b. Both 5,6α-EC and CES are involved in triglyceride accumulation (a characteristic of BC cell differentiation) and in cell death through an LXRβ-dependent mechanism. It is noteworthy that both 5,6α-EC and CES have been previously reported to be LXRβ modulators [60,61]. Furthermore, 5,6β-EC also participates in cell death induction through an LXRβ-independent mechanism. In contrast to sensitive MCF7 cells, MDA-MB-231 is resistant to the TAM inhibition of E2 mitogenic activity. Importantly, the ectopic expression of SULT2B1b in MDA-MB-231 cells increases the sensitivities of these cells with respect to the cytotoxic activity of TAM and the selective AEBS ligand, PBPE. In addition, MDA-MB-231 cells are as sensitive as MCF7 to the cytotoxic activity of CES (Figure 5XXI). The data presented above demonstrate that the CES/LXRβ axis, induced by AEBS ligands involving SULT2B1b expression, deserves to be considered as a mechanism of resistance against TAM. It is now evident that the mechanisms of TAM-induced cell death depend on the BC cell subtypes and are much more complex than a simple expression of the estrogen receptor. A transcriptomic study on several cell lines resistant to TAM has shown the involvement of a network of 38 genes of sterol and lipid metabolism due to an overexpression of Mucin 1 (MUC1) [111].

Altogether, TAM’s impact on cholesterol metabolism via the AEBS/ChEH/Sult2B1b/LXRβ pathway is a key mediator of the sensitivity and resistance of BC. The roles of several metabolites (5,6-EC, CES, and oncosterone), related enzymes (D8D7I, DHCR7, SULT2B1b, and 11β-HSD2), oxidative stress/antioxidant defenses (NRF2, SOD, Catalase, NADPH, and GSH), and nuclear receptors (LXR) deserve to be considered for the purposes of a better understanding of the intrinsic and acquired mechanism of resistance against TAM. The relationship between the ER status of BC and these metabolic pathways also remains unanswered. These events might convert ER-dependent BCs to hormone-independent human BC cells and thus lead to resistance to TAM.

## 6. Side Effects of Tamoxifen

The hormonal treatment of breast cancer is a procedure that drastically affects a patient’s quality of life. Due to complex drug regimens that are conducted in order to treat comorbidity conditions, older patients are more prone to adjuvant hormone intolerance [112]. Statistically, the tolerance toward hormone therapy among older BC patients ranges between 41% and 72% [113]. During greater doses of TAM, reactive oxidative species (ROS) are produced, which can have deleterious effects in not only BC cells, but also healthy cells. As a result, the release of ROS might lead to unintentional adverse effects under long-term therapy, including high toxicity and genotoxicity [40]. TAM induces a myriad of side effects, which include endometrial hyperplasia, polyps, fibrosis, cystic atrophy, and uterine sarcoma [114,115]. In a clinical study conducted on 204 patients aged between 27 and 84, after a 5-year period of TAM treatment, thromboembolic and uterine cancer mortality were only observed in women older than 55 [6]. In another random-effect meta-analysis report involving 53,000 women, the risk of endometrial cancer and of vascular and thrombotic events appeared to be less in premenopausal women [116]. Consequently, TAM did not increase the risk of endometrial cancer in premenopausal patients [117].

Patients might also experience cognitive disturbances known as TAM brain fog, which can result in effects such as decision-making impairment and the deterioration of executive functions [118,119], as well as depression [120]. Other complications encompass arthralgia, chills, night sweats, irregular heartbeat, insomnia, and hot flashes [121,122,123,124,125,126]. The long-term use of TAM might cause steatogenic hepatotoxicity in mice [127] and rats. In addition, TAM induced a liver iron overload, with unaltered hepatic function, in non-diabetic rats and might be a useful tool for investigating the biological control of iron metabolism [128]. The International Agency for Research on Cancer (IARC) rates TAM as being carcinogenic in experimental animals [129]. In a cross-sectional study of 32 women who were given TAM compared to 39 control women, TAM was reported to increase steatosis and adipose tissue distribution through its anti-estrogenic effect [130]. Massive hepatic steatosis in premenopausal women was also observed [131], suggesting that functional polymorphism in CYP17 is associated with circulating estrogen levels and is involved in basal hepatic lipid metabolism. An unsuitable effect of TAM on lipid metabolism includes hypertriglyceridemia [132,133], as well as severe acute pancreatitis in a dose-dependent manner [134]. Treatment with TAM was also correlated with clinical manifestations in the form of abnormal spotting or bleeding [115]. Other complications include congestion and sexual dysfunction [135]. In addition, gastrointestinal cancers [136] were also mentioned.

## 7. Insight into Vitamin E in Breast Cancer Chemo-Prevention

The prophylactic and therapeutic activities of vitamin E in BC have been exhaustively studied both in vitro and in vivo. Manifold research works have been performed beyond just investigating its antioxidant potential. Tocotrienols (TTs) and vitamin E esters have garnered ever-growing interest due to their patterns of specific activities. These metabolites possess potent chemo-preventive activities and are reported to modulate numerous intracellular signaling pathways and cellular processes. These include antiangiogenic potential, DNA and proteasome stabilization, proapoptotic activities, and cellular cycle arrest mediation. More particularly, TTs strongly suppress proliferation and promote apoptosis in cells [137,138,139,140,141,142,143,144,145,146,147,148,149,150,151,152,153].

In estrogen-responsive BC cells, TTs completely inhibit cell growth at a concentration of 8 µg/mL, whereas in estrogen-unresponsive cells, the complete suppression of cell growth was at 20 µg/mL [138]. TTs trigger apoptosis by mediating the mitochondrial death pathway [142] and several other oncogenic signaling pathways, such as TGF/SMAD and TRAIL. Unlike non-tumorigenic human MCF-10A cells, TTs, especially the delta congener, induce MDA-MB-435 and MCF-7 BC cells to undergo greater rates of apoptosis in a dose- and time-dependent manner. This mechanism involves the activation of TGF-β and Fas/CD95 signaling, upstream of the JNK phosphorylation pathway (TGF-β/Fas/JNK-signaling) [141]. For instance, γ-TT induces apoptosis in neoplastic mouse +SA mammary epithelial cells via the activation of endoplasmic reticulum stress markers, such as the PERK/eIF2alpha/ATF-4 pathway and the C/EBP homologous protein (CHOP) levels [148]. Moreover, γ-TT blocked tumor growth in a syngeneic implantation mouse cancer model and induced apoptosis via the activation of JNK and p38 MAPK, which up-regulate death receptor 5 (DR5) [150]. Apoptosis mediates a reduction in PI3K/PDK-1/Akt signaling [144,145,154]. PARP cleavage might also induce apoptosis in association with a decrease in the nuclear factor kappa-light-chain-enhancer of activated B cells (NF-κB) [155,156].

TTs also regulate NRF2-KEAP1 and induce the expression of cytoprotective oxidative stress modulatory genes, which consequently regulate proliferation in BC cells [157]. Increasing estrogen-responsive genes, such as early growth response-1 (EGR-1), cathepsin-D, and MIC-1, can lead to apoptotic alterations in cell morphology, DNA fragmentation, cell cycle arrest, and caspase-3 activation [146].

TTs are also potent inhibitors of cell proliferation, regardless of the ER status of cells. This can be attributed to their ability to down-regulate HMGR activity through the addition of mevalonolactone, a key metabolite in cholesterol biosynthesis. This finding highlights the importance of the isoprenoid–cholesterol pathway inhibition in this effect [153,158]. Moreover, TTs (delta form) induce cell cycle arrest in human BC cells associated with the loss of cyclin D1/ CDK4 expression [149,159].

In addition, γ-TTs are ERβ agonists and thus participate in a pro-apoptotic and cell growth inhibitory effect [146]. The form showed greater affinity for both receptors. In silico and in vitro experiments on MDA-MB-231—which expresses only Erβ—and MCF-7—which expresses two ER isoforms—showed that the different forms of tocols acted as modulators rather than pure agonists [153]. In murine mammary cancer cells, TTs prevent angiogenesis by increasing interleukin-24 (IL-24) mRNA expression and decreasing IL-8 and vascular endothelial growth factor mRNA levels [160]. In animal studies, it was reported that there was only marginal chemo-preventive activity present against the DMBA-induced tumors of tocotrienol and tocopherol [161,162]. A similar experiment conducted by Iqbal et al. (2003) reported the suppression of 7,12-dimethylbenz[alpha]anthracene-induced carcinogenesis in rats fed a tocotrienol-rich fraction isolated from rice bran oil. This finding was also associated with low serum cholesterol and low-density lipoprotein (LDL) concentrations [163].

Other reports of studies in vivo support evidence of a decrease in tumorigenesis with high-vitamin-E diets. TTs were shown to inhibit proliferating cell nuclear antigen (PCNA) in *N*-methyl-*N*-nitrosourea-induced mammary hyperplasia in female Sprague Dawley rats [164]. Furthermore, unlike α-TP, other congeners inhibit mammary tumorigenesis in animals [165]. More particularly, in female Sprague Dawley rats treated with a single intraperitoneal injection of carcinogenic *N*-methyl-*N*-nitrosourea (NMU, 50 mg/kg body mass)—unlike α-TP which did not reduce tumor multiplicity in a dose-dependent manner—the other congeners revealed a decrease in the tumor. This can be attributed to the activation of cyclin-dependent kinase (CDK) inhibitors p21, p27, and caspase-3, and peroxisome proliferator-activated receptor-γ (PPAR-γ) [166].

Although they possess strong chemo-preventive properties, TTs and other congeners of tocopherols have much lower systemic bioavailability than the free form of α-TP [167]. Several formulation strategies have already been designed to circumvent the poor oral absorption of TTs, as well as to improve their antiproliferative activities in vitro and tumor suppression in vivo. These include self-emulsifying delivery systems, nanostructured lipid carriers [168], intravenous injections of polymer-conjugated tocotrienols [169], nanovesicles [170], and nano-emulsions for topical applications [171].

**Figure 6:**
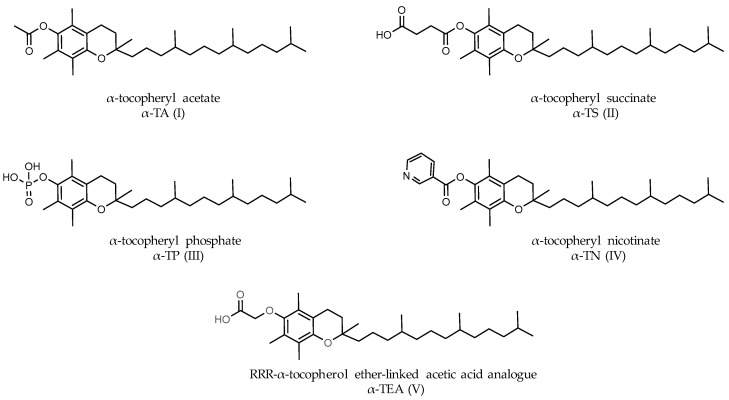
The structures of vitamin E esters.

Certain semi-synthetic analogs of vitamin E are commercially available as redox-silent congeners. These often include esters such as α-tocopheryl acetate (α-TA, Figure 6I) and α-tocopheryl succinate (Figure 6II). Few data were, however, observed for other esters, such as α-tocopheryl phosphate (Figure 6III) and α-tocopheryl nicotinate (Figure 6IV), which are esters of vitamin E and niacin. The most studied was α-tocopheryl succinate (α-TS), while α-tocopheryl acetate (α-TA) and its stable ether-linked acetic acid analog (α-TEA, Figure 6V) were less studied. Vitamin E ester forms are also prone to hydrolysis after oral administration. The cleavage by cellular esterases releases the free forms, which are less active as anticancer agents, and hence might represent a limiting factor. Synthetically produced α-TEA with a non-hydrolyzable ether linkage remained intact in the cell compared to its ester-linked derivatives [172]. α-TEA and α-TS are potent adjuvants in cancer chemo-prevention and treatment. They showed distinct apoptogenic effects in a concentration- and time-dependent manner within several cancer cells [141,172,173,174,175,176,177]. With α-TEA, certain reports showed evidence of apoptosis through endoplasmic reticulum stress-mediated JNK/CHOP/DR5/caspase-8 signaling [178] and the IRS/PI3K pathway, which serves as a potential therapeutic target [179].

Dietary exposure to 3.3 g α-TEA/kg, which was approximately 5 mg/mouse/day, significantly suppressed the growth of 4T1 mammary tumors in mice [180]. Exposure to α-TEA reduces oncogenic human epidermal growth factor receptor 2 (HER2/neu) expression and enhances the antitumor immune response [181]. Cell surface components associated with the death-receptor-mediated pathways of α-TEA-induced apoptosis include the TGF-β type II receptor I [141]. The JNK/p73/NOXA gene axis was seen to be up-regulated in association with a decrease in anti-apoptotic mediators, including AKT, ERK, c-FLIP, and survivin [182,183].

Furthermore, α-TS (Figure 6II) has been the source of numerous exhaustive studies and has potentially superior biological activities in comparison to free forms of tocopherols and other α-tocopherol esters. Unlike the potent hydrolytic conversion of α-TS to its α-tocopherol free form in normal cells, cancer cells exhibit low hydrolytic activity with respect to α-TS [174,184]. Moreover, a weak cell transmembrane pH gradient in tumors can help to enhance the cytotoxicity of specific weak acidic properties of α-TS [185]. In addition, α-TS actions are also rooted in a considerable diversification of signal transduction pathways and the levels of gene expression [174,175,184,186]. More particularly, they induce DNA synthesis arrest [187], inhibit metastasis [188], and block angiogenesis [189].

Moreover, α-TS is a potent pro-apoptotic agent that restores the transforming growth factor-beta (TGF-β) and Fas (CD95) apoptotic signaling pathways, which contribute to JNK-mediated apoptosis and TRAIL/DR5 death receptor pathway activation [190]. Furthermore, α-TS mediates cell cycle arrest and differentiation via up-regulating the cyclin-dependent kinase inhibitor p21 (Waf1/Cip1) and inhibiting the release of the angiogenic peptide vascular endothelial growth factor-A [187,189,191]. Furthermore, it also inhibits both the human epidermal growth factor receptor and the antiapoptotic factors [143,176,178,183,192]. Additionally, the apoptogenic effect was bolstered by the inhibition of the pleotropic nuclear factor-kappa B (NF-κB) and a decrease in the levels of interleukin and vascular endothelial growth factors [193]. It has been also suggested that the mechanisms leading to α-TS-induced apoptosis in the MDA-MB-231 cell line share the activation of a common pathway, which involves NF-κB inhibition, as well as caspase-8 activation and Fas/Fas L stimulation [177].

In another study that included PKC inhibition via protein phosphatase-2A activation in BC cell lines (MCF-7 and MDA-MB-435), neither tocopherols nor their acetate esterified forms were apoptogenic [173]. Moreover, α-TS activates the other tumor necrosis factor (TNF) receptors, such as the first immunogenic apoptosis signal death receptor (Fas) [176]. Additionally, α-TS, unlike its free form, selectively induces apoptosis in tumor cells by disrupting mitochondria and forming reactive oxygen species (ROS), which subsequently activate Bcl-2-associated X protein channels, thus allowing for caspase-3 and -9 activation and apoptosis [173,194,195,196,197,198].

Other mechanisms have been also described, such as disrupting the structural integrity of the inner mitochondrial membrane under the detersive action of α-TS and its amphipathic structure [174]. In addition, α-TS specifically suppresses tumor growth via the interference of the mitochondrial complex II, as well as through sphingomyelinase activation [189,196,197,199].

To overcome the issues of the bioavailability and stability of α-TS, liposomal, nanoparticular, and micellar formulations have been designed. Examples include tocopheryl succinate-nanovesicles (α-TS-NV) combined with egg phosphatidylcholine (α-TS-EPC-NV) and the esterification of α-TS with polyethylene glycol [200,201].

Certain authors have suggested a limited potential of clinical support for vitamin E in chemo-prevention, as conflicting results in the epidemiological studies of its effectiveness have been reported [202,203]. The study designs were generally cohort studies, as well as case–controls, randomized controlled trials, and nested case–control studies. Certain studies noted the importance of the congener type for BC, either in pre- or post-menopausal women. Others found some sort of correlation between vitamin E and BC risk reduction [204,205,206,207,208,209,210,211,212,213]. Additionally, some clinical studies even noted increased cancer mortality among the long-term antioxidant consumers, along with chronic diseases and poor health status [214,215].

## 8. Tamoxifen and Vitamin E Inhibitory Effects

There are numerous concerns that persist regarding the risk/benefit ratio of dietary antioxidant supply during cancer treatment. Bioavailability, antioxidant status, and genetic polymorphisms might interfere with and further complicate medical care [216]. TAM has been involved in coordinating several pathways and causing side effects. Combinatory treatments have been introduced to help to diminish the adverse effects of TAM. Certain drugs, such as the Cox-2 inhibitor celecoxib, enhance TAM-mediated tumor growth inhibition in ER-positive BC cells. At non-toxic doses, a combination of TAM and celecoxib results in anti-angiogenic effects via the specific targeting of VEGF/VEGFR2 autocrine signaling [217]. Everolimus, an inhibitor of PKC and mTOR, also showed a significant clinical benefit when combined with TAM, compared to TAM alone [218].

An example of a natural product that helps to enhance TAM therapy and ameliorate its cytotoxicity is carnosic acid. When combined with TAM, it induces caspase-3-activated apoptosis in BC cells [219]. Other products of natural origin include equol [220], the phospholipid complex [221], poly(D, L-lactic acid) [222], thymoquinone [223], caffeic acid phenethyl ester [224], xanthene hybrid [225], lycopene [226], lauryl gallate [227], and zinc [228]. It has been recently reported that the combination treatment of TAM with poly-botanical dietary supplements and their extracts enhances its anticancer properties and helps to increase the sensitivity of TAM-resistant cell lines [229]. Additionally, in postmenopausal BC patients who took antioxidant-supplemented drugs—such as coenzyme Q(10), niacin, and riboflavin—for 90 days, TAM induced an increase in antioxidant status and a decrease in oxidative stress, plasma lipids, and lipid peroxides [230]. In an animal study of DMBA-induced mammary carcinogenesis in Sprague Dawley rats, treatment with TAM was the most effective when combined with riboflavin, niacin, and CoQ10 by restoring lipid peroxide levels and enhancing antitumor activities in isolated mammary gland mitochondria [231].

Vitamin E might have different anticancer effects through undefined mechanisms when used in combination with different chemical agents. In certain in vitro reports, a multicomponent approach associating TTs with other natural or synthetic chemo-preventive agents was recognized as a more effective chemo-preventive strategy for BC when compared to monotherapy. When combined with TAM, TT-rich fraction (TRF) from palm oil, as well as δ and γ-TT, was more effective against ER-negative MDA-MB-435 and ER-positive MCF-7 human BC cells. As such, this respective synergistic effect could be considered to be an improvement in BC therapy [232]. However, according to certain reports, the concurrent treatment of vitamin E supplementation during cancer treatment made it difficult to obtain and sustain therapeutic levels in target tissues; in addition, it was even reported to be harmful [10,203,233]. In an in vitro study, it was reported that vitamin E not only scavenges ROS, but also significantly promotes MCF-7 cell proliferation by reducing ROS production and down-regulating the expression of tumor suppressor p53 [234]. A few other in vitro studies have also supported the hypothesis that vitamin E abrogates TAM’s effects [51,109,110,112,113,235,236].

Vitamin E appears to alter TAM-induced cell growth inhibition in the ER-positive BC cell lines of MCF-7 and T47D. In MCF-7 cells, vitamin E (100 µM) with 5–10 µM of TAM reduced growth inhibition by 20%. Vitamin E decreased the growth inhibition induced by TAM in MCF-7 by 33% and in T47D by 54%. This raises some concerns that α-TP supplementation might reduce the effectiveness of TAM therapy [235]. In Peralta et al.’s report (2006), supplemental vitamin E acetate (Figure 6I) (50 µM) decreased the inhibitory effect of a TAM-induced rise in intracellular Ca2+ in MCF-7 and T47D cells. They also noted a restoration of cell proliferation and p-ERK expression in a dose-dependent manner, decreasing apoptosis even in the presence of a high dose of TAM [236]. Three years later, according to Peralta et al. (2009) in their prospective study, it was suggested that vitamin E supplementation might interfere with the subtherapeutic blood levels of TAM. As a matter of fact, TAM blood levels decreased in the biomarkers of estrogen stimulation in breast biopsies (estrogen receptors, progesterone receptors, and pro-surviving signaling p-ERK) [237]. However, due to the limited number of patients (only seven) included in this study, valid conclusions regarding vitamin E and TAM interaction need to be induced by large, randomized clinical trials.

Growth-factor receptors, including total and phosphorylated forms of HER-1 and HER-2, as well as their downstream pro-survival mediators and cholesterol-rich lipid microdomains, were highly expressed in TAM-resistant cell lines. Tiwary et al. (2011b) reported that the targeting of cholesterol-rich lipid microdomains is a potent strategy to overcome resistance to TAM in BC cell lines. More particularly, a combination of α-TEA (Figure 6V) and TAM might circumvent resistance to TAM through the suppression of pro-survival signaling, the disruption of cholesterol-rich lipid microdomains, and the induction of endoplasmic reticulum stress-triggered death via the pro-death of the pJNK/CHOP/DR5 amplification loop [238].

A double-blinded, controlled clinical trial was conducted with 240 women aged 40–60, with either early stage I or II estrogen-receptor-positive BC. Adjuvant TTs, given in combination with TAM, were found to not be protective in regard to BC-related mortality and recurrence in patients when compared to TAM alone. Combinations of TTs (400 mg/day) and TAM (20 mg) each day for a period of 5 years in a double-blinded, placebo-controlled pilot trial resulted in an increase in vitamin E blood levels and normal liver function. Moreover, TAM intake alone was found to not be statistically significant in confirming any synergistic effect of tocotrienols [139,140]. In another reported work [12], it was noted that no evidence from animal models or randomized controlled human trials suggests any association between the intake of TPs and TTs at nutritionally relevant doses. However, large doses of vitamin E, such as those greater than 300 mg/d, might lead to interactions with drugs such as TAM. Although TAM activity was reduced in the presence of vitamin E in a cellular model, there are still no in vivo studies to support these findings.

Discrepancies in results concerning the pharmacogenomics of TAM have been reported. Therefore, the intensity and duration of TAM treatment should be associated with each patient’s individual genetic predisposition. One of the most susceptible genes associated with BC is the catechol-*O*-methyltransferase (COMT) gene. COMT, a methyl donor phase II enzyme involved in the detoxification of xenobiotics and estrogens, is associated with a wide variety of cancers, including BC [239]. As a result, different responses to the anti-estrogen treatment with different outcomes might be possible.

## 9. Conclusions

Clinicians should cautiously guide patients against vitamin E during cancer treatment with TAM, pending more recommended clinical trials. Therefore, vitamin E should not be recommended to patients undergoing TAM therapy, especially if they are not deficient in vitamin E. Considering the pharmacology of tocols, it is now recommended to specify which free congeners or esterified forms are being studied for any future clinical trial. A more substantial conclusion has yet to surface, and other proposed therapies might consist of a further revision of drug cocktails, which, in turn, might affect different signals synchronously.
